# *De Novo* Transcriptome Assembly and Comparative Analysis Elucidate Complicated Mechanism Regulating *Astragalus chrysochlorus* Response to Selenium Stimuli

**DOI:** 10.1371/journal.pone.0135677

**Published:** 2015-10-02

**Authors:** Özgür Çakır, Neslihan Turgut-Kara, Şule Arı, Baohong Zhang

**Affiliations:** 1 Department of Molecular Biology and Genetics, Faculty of Science, Istanbul University, 34134 Vezneciler Istanbul, Turkey; 2 Research and Application Center for Biotechnology and Genetic Engineering, Istanbul University, 34134, Istanbul, Turkey; 3 Department of Biology, East Carolina University, Greenville, NC 27858, United States of America; National Key Laboratory of Crop Genetic Improvement, CHINA

## Abstract

*Astragalus* species are medicinal plants that are used in the world for years. Some *Astragalus* species are known for selenium accumulation and tolerance and one of them is *Astragalus chrysochlorus*, a secondary selenium accumulator. In this study, we employed Illumina deep sequencing technology for the first time to *de novo* assemble *A*. *chrysochlorus* transcriptome and identify the differentially expressed genes after selenate treatment. Totally, 59,656 unigenes were annotated with different databases and 53,960 unigenes were detected in NR database. Transcriptome in *A*. *chrysochlorus* is closer to *Glycine max* than other plant species with 43,1 percentage of similarity. Annotated unigenes were also used for gene ontology enrichment and pathway enrichment analysis. The most significant genes and pathways were ABC transporters, plant pathogen interaction, biosynthesis of secondary metabolites and carbohydrate metabolism. Our results will help to enlighten the selenium accumulation and tolerance mechanisms, respectively in plants.

## Introduction

*Astragalus* species have hepatoprotective, antioxidative, immunostimulative and antiviral properties and because of these properties they are being consumed in the world [1]. These properties also led to efforts to illuminate chemical contents of *Astragalus* plants and they have gained economical and medicinal values [2, 3]. *Astragalus* genus is a member of Leguminosae and also is famous for accumulation of high levels of selenium [4, 5]. *Astragalus bisulcatus* is a well-studied plant that accumulates selenium in high level as well as 0.65% in its tissues [6]. Among the *Astragalus* species, the Turkish endemic *A*. *chrysochlorus* has been proved to be one of secondary selenium accumulators [7]. In our previous study, SMT (selenocysteine methyltransferase) which is known to be an essential enzyme in Se (selenium) metabolism, has been isolated from *A*. *chrysochlorus* [8]. Additionally, the potential roles of microRNAs (miRNAs) in selenium stimuli were investigated in *A*. *chrysochlorus* [9].

It is known that most plants can not tolerate high levels of selenium. However, some plants can tolerate high concentrations and accumulate Se. Thus, it could be toxic for most of the plants. Selenium is known to be taken into cells by sulphate transporters because it resembles sulfur (S) and therefore it is metabolized in sulfur-related pathways. For bioremediation and biofortification studies, it is of necessity to know the details of accumulation of high levels of selenium and underlying mechanism. Se transportation and accumulation has become the focus of interest recently [10–14]. There is the necessity of new approaches that will enlighten the mechanisms underlying Se tolerance and accumulation. Identification of the genes and their expression profiling will help us to understand the mechanisms. Next generation sequencing is one of the strategies that could be used to investigate the transcriptome of a non-model plant in a certain condition. RNA-seq technologies provide a fast and cheaper solution to study the genetic information that organisms contain and this method is functional in non-model plant species when the genome information is unavailable, and the RNA-seq deals with the coding regions not the whole genome [15]. Currently, there is no report on deep sequencing assembly and genome-wide expression profile in *A*. *chrysochlorus*. This limits the application of *A*. *chrysochlorus* in biomedical field as well as the genetic improvement of this plant species for agricultural and medical purpose.

In this study, we aimed to identify essential genes and their expression under Se treatment. The RNA-seq was done to assemble *de novo* and annotate the transcriptome of *Astragalus chrysochlorus*. Bioinformatic tools were applied to investigate the pathways and genes that were expressed under Se treatment for better understanding Se tolerance and accumulation.

## Materials and Methods

### Plant material and culturing conditions

The callus cultures of *Astragalus chrysochlorus* used in this study were established and maintained as long-term previously [16]. Callus tissues were subcultured every three weeks. For selenium treatment, 21-day-old callus tissues were grown on MS medium supplemented with 5 mg/L sodium selenate for 3 weeks. Tissue culture tests were done using a growth chamber with fluorescent light illumination (ca. 1400 mol -2 ms -1) over a 16/8 day and night at 25± 2°C. Control and selenium treatments were triplicated in five individual culture dishes, and each dish contained nine callus tissues. After 21 days, callus tissues were harvested and immediately frozen in liquid nitrogen and then stored in -80°C for RNA extraction.

### RNA Isolation

Total RNA was extracted using Qiagen total RNA isolation system (RNeasy Plant Mini Kit, 74904, Qiagen) according to the manufacturer's protocol. RNAs quality and quantity were checked using a Nanodrop 2000 spectrophotometer (Nanodrop Technologies, USA). Total RNA was then used for library preparation and sequencing using Illumina HiSeq 2000 sequencing.

### Library Preparation and Sequencing

mRNA isolation, fragmentation, adapter ligation, cDNA library construction and sequencing were done by Beijing Genome Institute (BGI) (Shenzhen, China). Briefly, after checking the RNA quality and quantity, the mRNA was isolated using magnetic beads, then mRNAs were fragmented using fragmentation buffer. These fragments were used as templates for cDNA synthesis. Single nucleotide A (adenine) was added to obtain short fragments. Adapters were ligated to short fragments, then PCR was performed. Illumina HiSeq 2000 was used to sequence the libraries.

### Sequencing Analysis

After the deep sequencing, raw reads with low quality were first eliminated. Transcriptome assembly of short reads were done by Trinity program (release-20130225, http://trinityrnaseq.sourceforge.net/) [17] operating with three individual modules (Inchworm, Chrysalis and Butterfly). These three softwares were run consecutively. The parameters for Trinity were—seqType fq—min_contig_length 100,—min_glue 3—group_pairs_distance 250 and—path_reinforcement_distance 85—min_kmer_cov 3. The contigs that were obtained, joined together to get scaffolds. Then, the Gene Indices Clustering Tools (TGICL, version 2.1) [18] were used to form unigenes and Phrap (Release 23.0) (http://www.phrap.org/) was used to assemble scaffolds to cluster the scaffolds [19]. After all, assembled transcriptome sequences of *Astragalus chrysochlorus* were obtained. The unigenes were subjected to blastx alignment (evalue < 0.00001) in NR, Swiss-Prot, KEGG and COG databases. The results from these aligments were used to decide the direction of sequences. Lastly, the sequences which do not be aligned to any database were subjected to ESTScan [20]. For function annotation, Blast was used (v2.2.26+x64-linux) against NT (NCBI nucleotide database, release-20130408), NR (non-redundant protein sequence database, release-20130408), KEGG (Kyoto Encyclopedia of Genes and Genomes, Release 63.0), Swiss-Prot (release-2013_03) and COG (Clusters of Orthologous Groups, release-20090331) databases. To determine the unigene GO annotation, NR annotation was used. Blast2GO (http://www.blast2go.com/b2ghome, release 2012-08-01)[21] program was run to get the gene ontology. Expression profiling of unigenes were done. The reads were mapped to reference transcriptome by SOAP (Release 2.21, http://soap.genomics.org.cn/soapaligner.html), using the parameters “-m 0-x 500-s 40-l 35-v 5-r 1”. FPKM (Fragments Per kb per Million reads) method was done. With this method, it is possible to eliminate the effect of different gene length. Thus, it is possible to compare the expression level of a gene in different samples. RPKM was calculated according to the unigenes length and read number mapped to this gene [22]. Statistical analysis was done to compare different libraries. False discovery rate (FDR) was calculated to specify the p-value threshold in expression analysis. If the FDR is smaller and the fold change is bigger, it means that the expression difference between the two samples is bigger. The criteria were used as FDR ≤0.001 and fold change (Se treated/not treated) ≥ 1 or ≤ -1. Lastly, GO and KEGG pathway analysis were done for the differentially expressed genes according to the description above.

## Results

**Fig 1:**
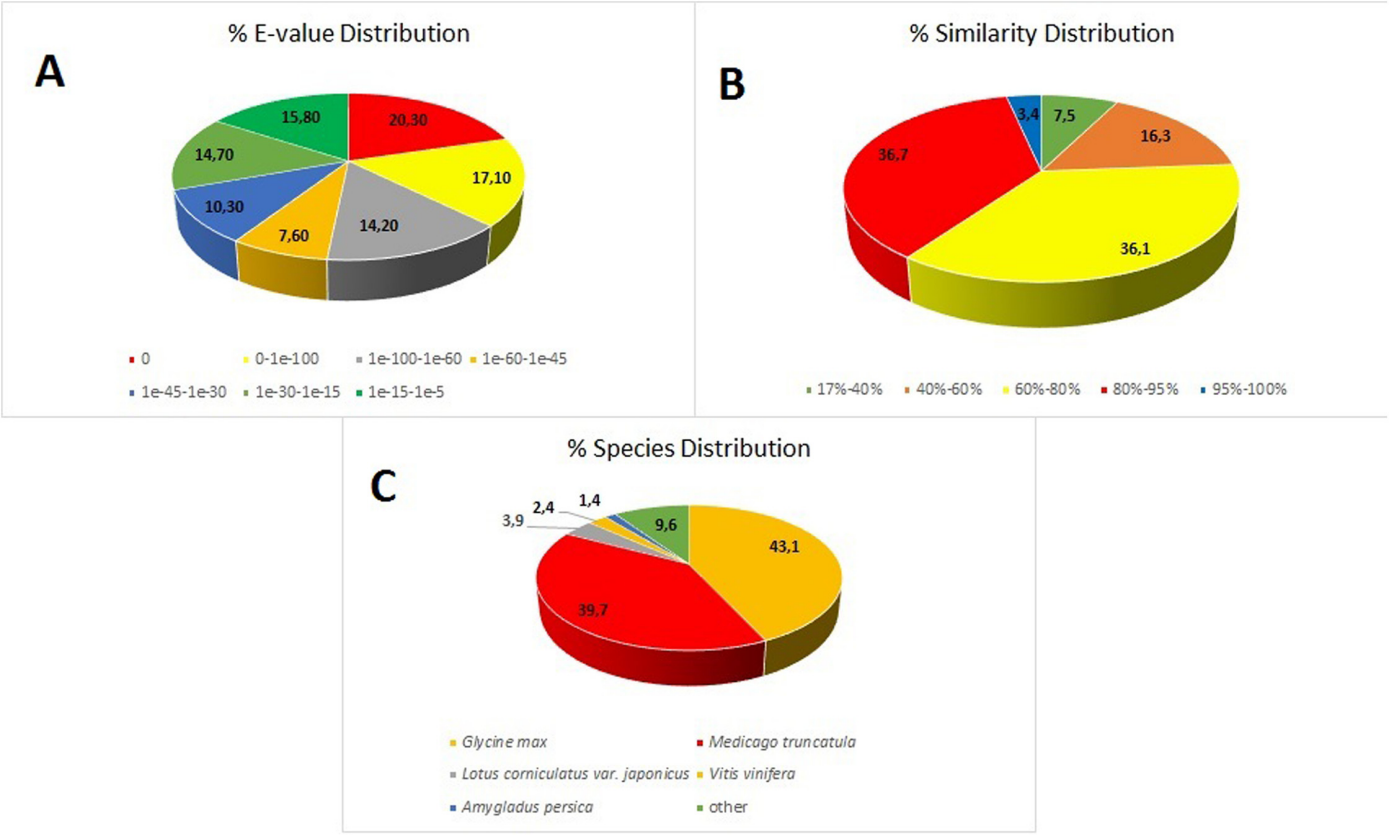
Unigene annotation results according to the NR database. (A) The E-value distribution. (B) The similarity distribution. (C) The species distribution.

The raw data of *A*. *chrysochlorus* RNA-seq were deposited in NCBI Gene Expression Omnibus (GEO) database (accession number: GSE71097). We obtained a total of 108,123,354 raw reads from two libraries (control and selenium-treated) using paired-end sequencing on Illumina Hiseq 2000 platform. Totally, 9,358,590,780 clean nucleotide reads were generated from two libraries. From these reads, a total of 83,273 unigenes were assembled and identified with a total of 93,072,751 nt in length with an average of 1,118 nt. Then the numbers of unigenes were determined via function annotation analysis and length distribution sizes of *A*. *chrysochlorus* unigenes were calculated (Table 1 and S1 Fig). A total of 52,640 CDS were mapped to protein database. Total raw reads for control and Se-treated samples were similar with 53,984,240 and 54,139,114, respectively (Table 2). It was determined that the clean reads were more than 95% in both samples. An average of 658,976-fold of sequencing depth was obtained (**S1 and S2 Tables**). The Q20 value of the reads were over 98% for both samples. **Fig 1** summarizes the e-value similarity and species distribution. According to the NR database similarity search, it was found that 59.2 of the sequences showed high degree of similarity (<1e^-45^), 40.8% of the sequences showed reasonable similarity (between 1e^-5^ and 1e^-45^). According to the similarity distribution, 40.1% of the sequences showed more than 80% similarity with the sequences in the database.

**Table 1:** Annotation results of *A*. *chrysochlorus* unigenes according to different databases.

Database	Number of Unigene	Percentage
**NR**	53,960	90
**NT**	54,521	91
**Swiss-prot**	33,650	56
**KEGG**	31,003	51
**COG**	19,318	32
**GO**	40,599	68

**Table 2:** Sequencing statistics of Se-treated and untreated *A*. *chrysochlorus* callus tissues.

Samples	Total Raw Reads	Total Clean Reads	Total Clean Nucleotides (nt)	Q20 percentage	N percentage	GC percentage
**Control**	53,984,240	51,945,966	4,675,136,940	98.54%	0.00%	40.96%
**Se-treated**	54,139,114	52,038,376	4,683,453,840	98.49%	0.00%	41.74%

**Fig 2:**
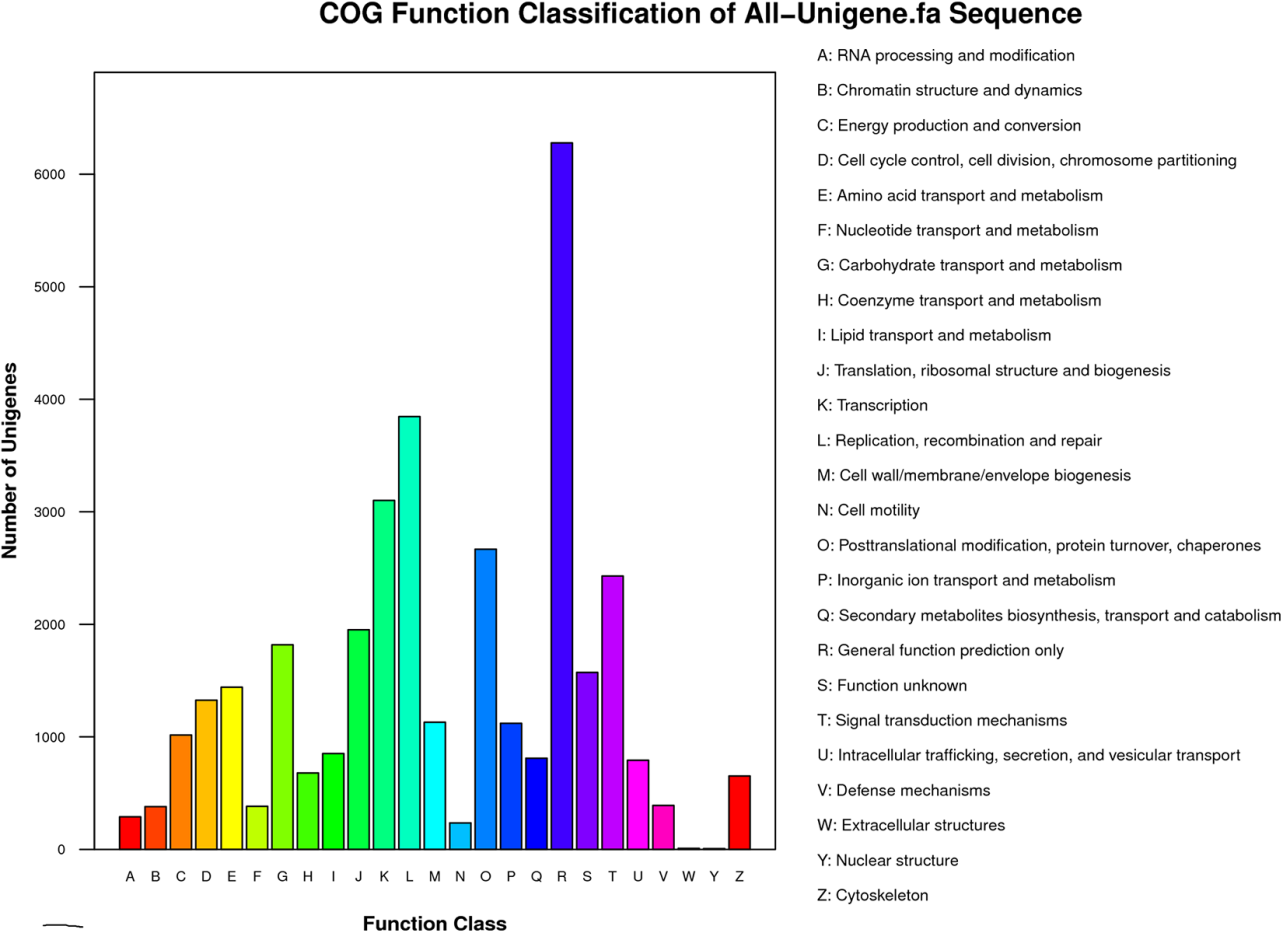
Functional classification of *A*. *chrysochlorus* unigenes according to COG database.

**Fig 3:**
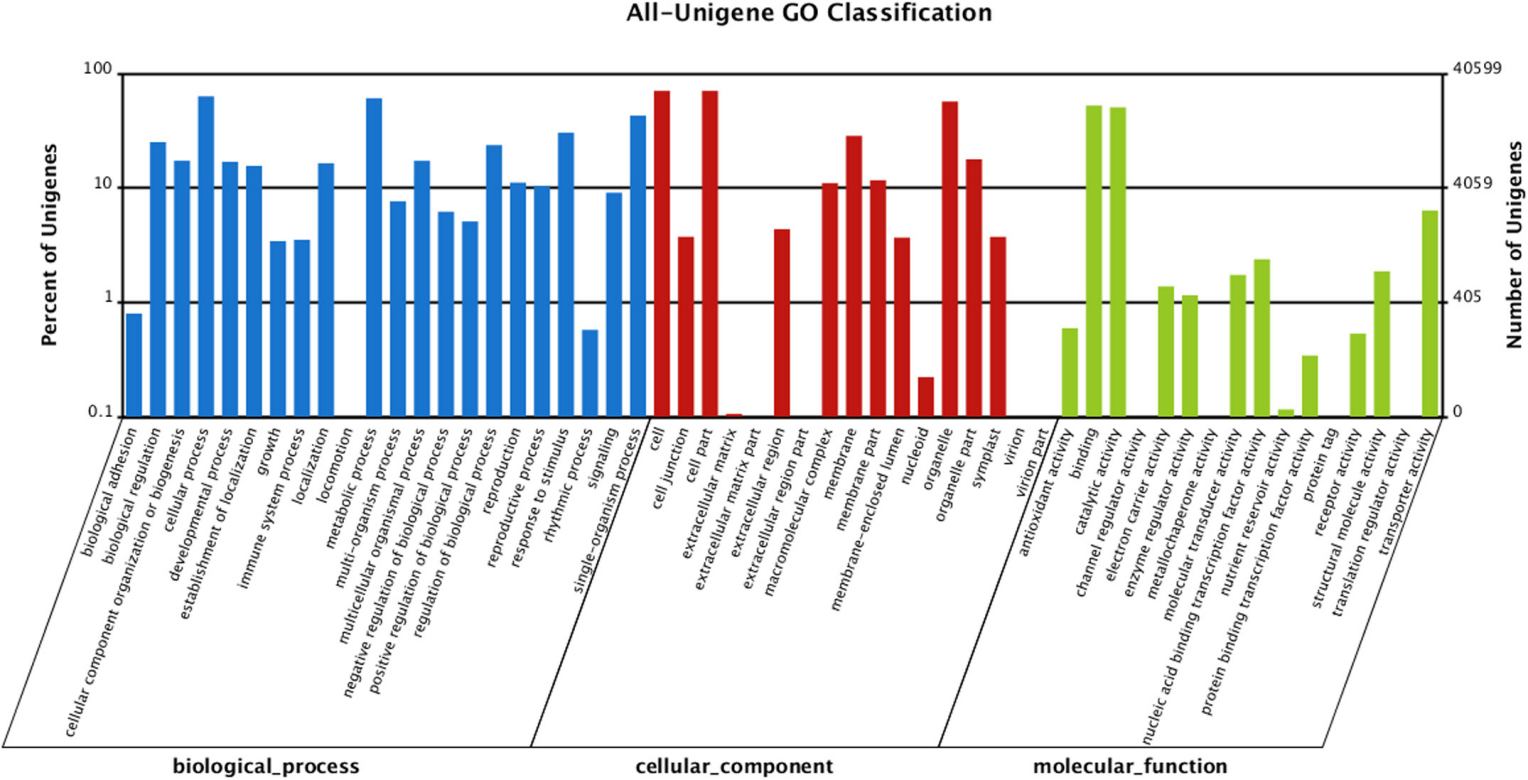
GO functional classification of *A*. *chrysochlorus* unigenes.

**Fig 4:**
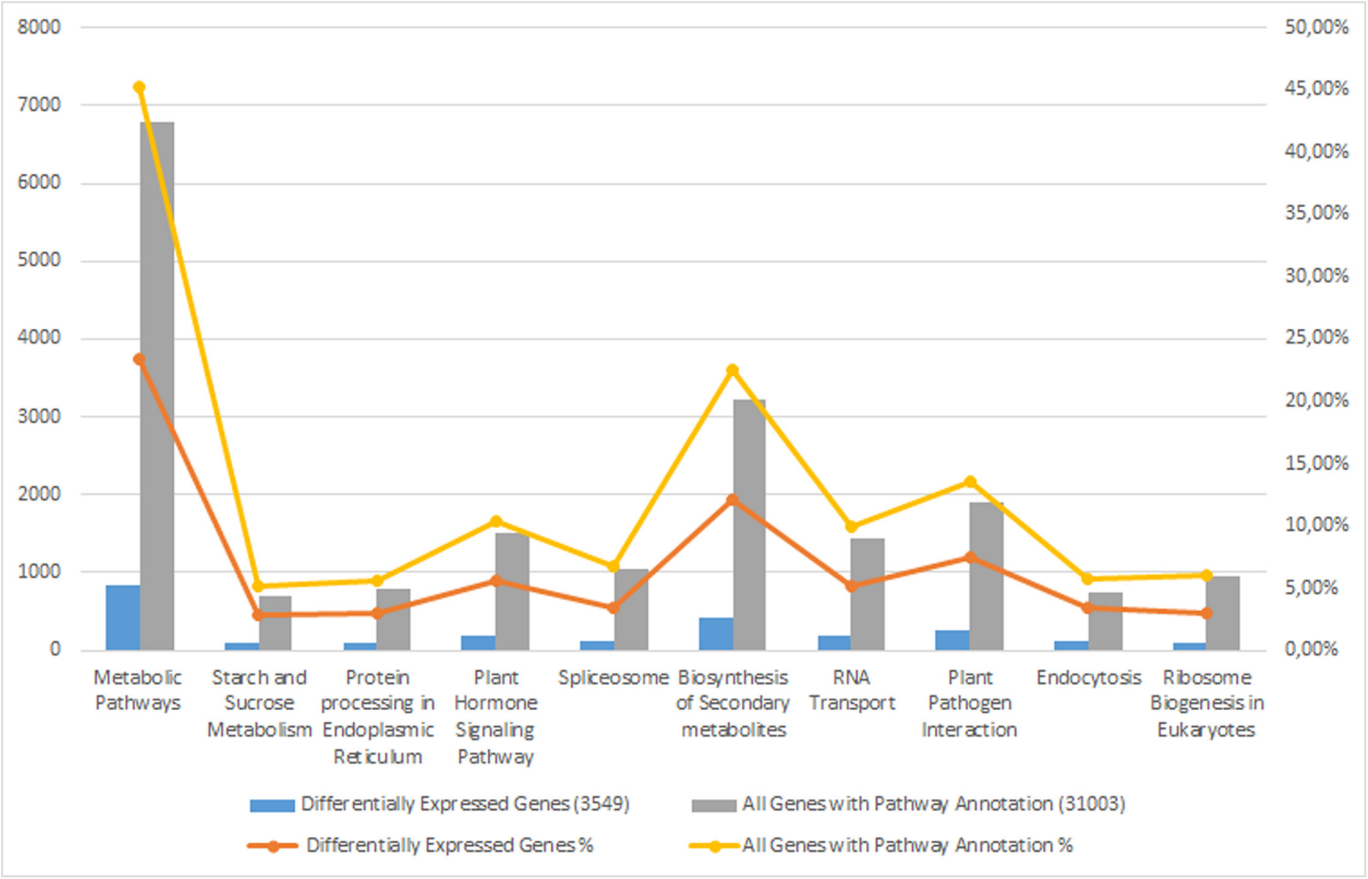
KEGG pathway annotation of unigenes related with Se-treated and untreated *A*. *chrysochlorus* calli metabolism.

We also compared the transcriptome similarity between *A*. *chrysochlorus* and other closely-related plant species. A total of 43.1% of *A*. *chrysochlorus* transcriptome were matched to *Glycine max* (43.1%) followed by *Medicago truncatula* (39.7%) and *Lotus corniculatus* var. *japonicus* (9.6%), respectively. For the COG classification, a total of 35,175 genes were categorized into 25 classes (**Fig 2**). According to the number of genes, the most significant ones were general function (6,278), replication, recombination and repair (3,846), transcription (3,101), posttranslational modification, protein turnover and chaperones (2,668) and signal transduction mechanisms (2,429). According to the Gene ontology analysis, unigenes were sorted into 55 categories (**Fig 3**). In biological process category, cellular process, metabolic process and single cell process ontologies were the top three gene ontology terms with the number of unigenes 25,641, 24,683 and 17,416, respectively. In the same category, biological adhesion, ryhtmic process and locomotion were the last three GO terms with the number of unigenes 324, 233 and 31, respectively. In cellular component category, cell, cell part and oganelle terms were the top three classes with 28,623, 28,622 and 23,094 unigenes. The last three categories were extracellular matrix part, extracellular region part and virion and virion part with the number of 26, 17, and 18, respectively. Lastly, for molecular function category, binding, catalytic activity and transporter activity were the top three GO terms with the number of unigenes 21,302, 20,560 and 2,578, respectively. The categories with the least three unigene numbers were metallochaperone activity (6), translation regulator activity (4) and protein tag (4), respectively. For KEGG analysis, 31,003 unigenes were annotated with the pathway analysis and it is determined that 3,549 of them were differentially expressed. These unigenes were annotated with 127 pathways according to KEGG analysis. The most significant unigene numbers that were detected, were listed in **Fig 4.** Metabolic pathways are the first category with 828 differentially expressed unigenes. Biosynthesis of secondary metabolites and plant-pathogen interaction are the second and third categories with 429 and 263 differentially expressed unigenes, respectively.

**Fig 5:**
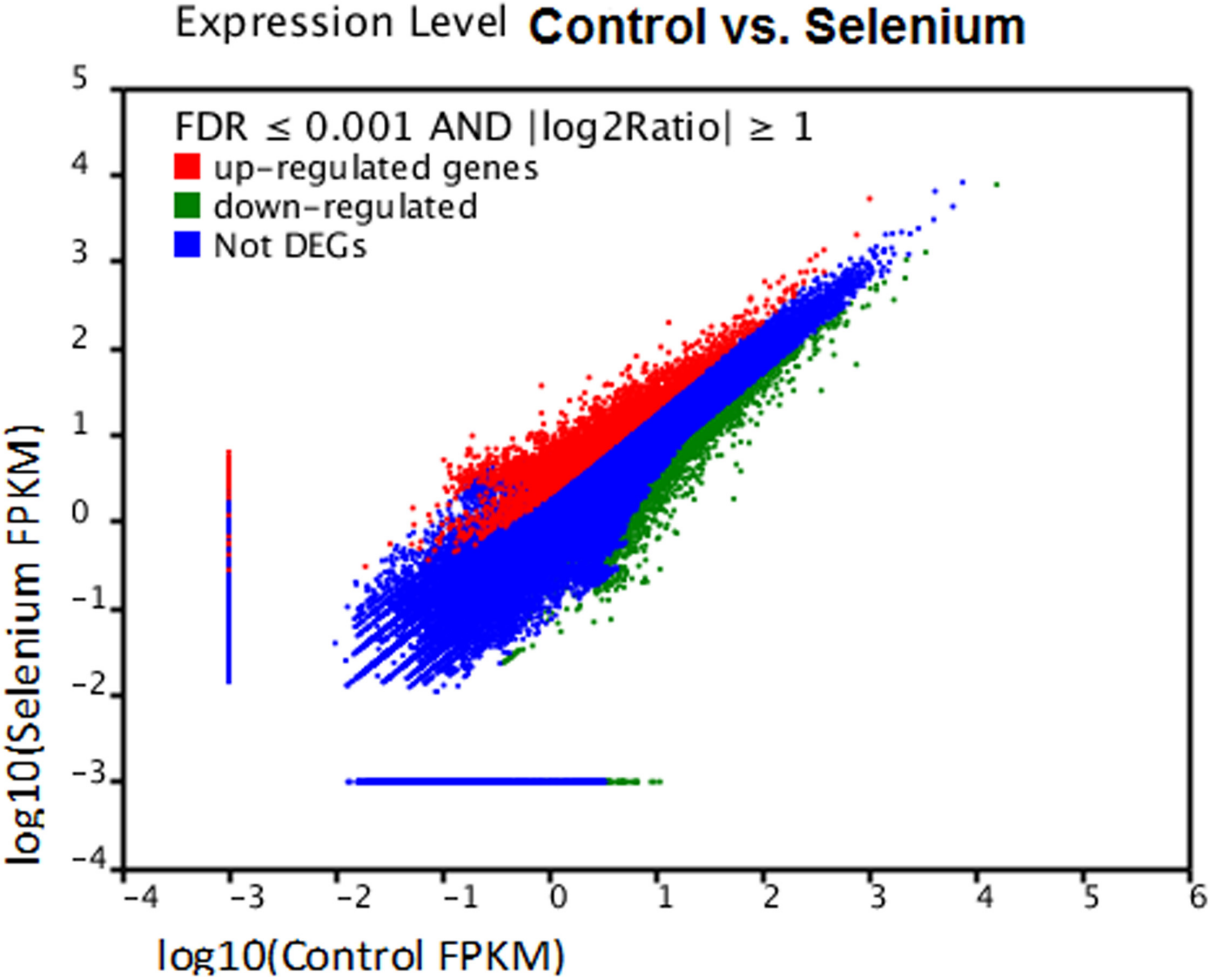
Differential expression level of Se-treated and untreated *A*. *chrysochlorus* callus tissues. Red and green colours represent up-regulation and down-regulation of Se-treated vs. control genes, respectively. Blue colour means not differentially expressed genes.

**Fig 6:**
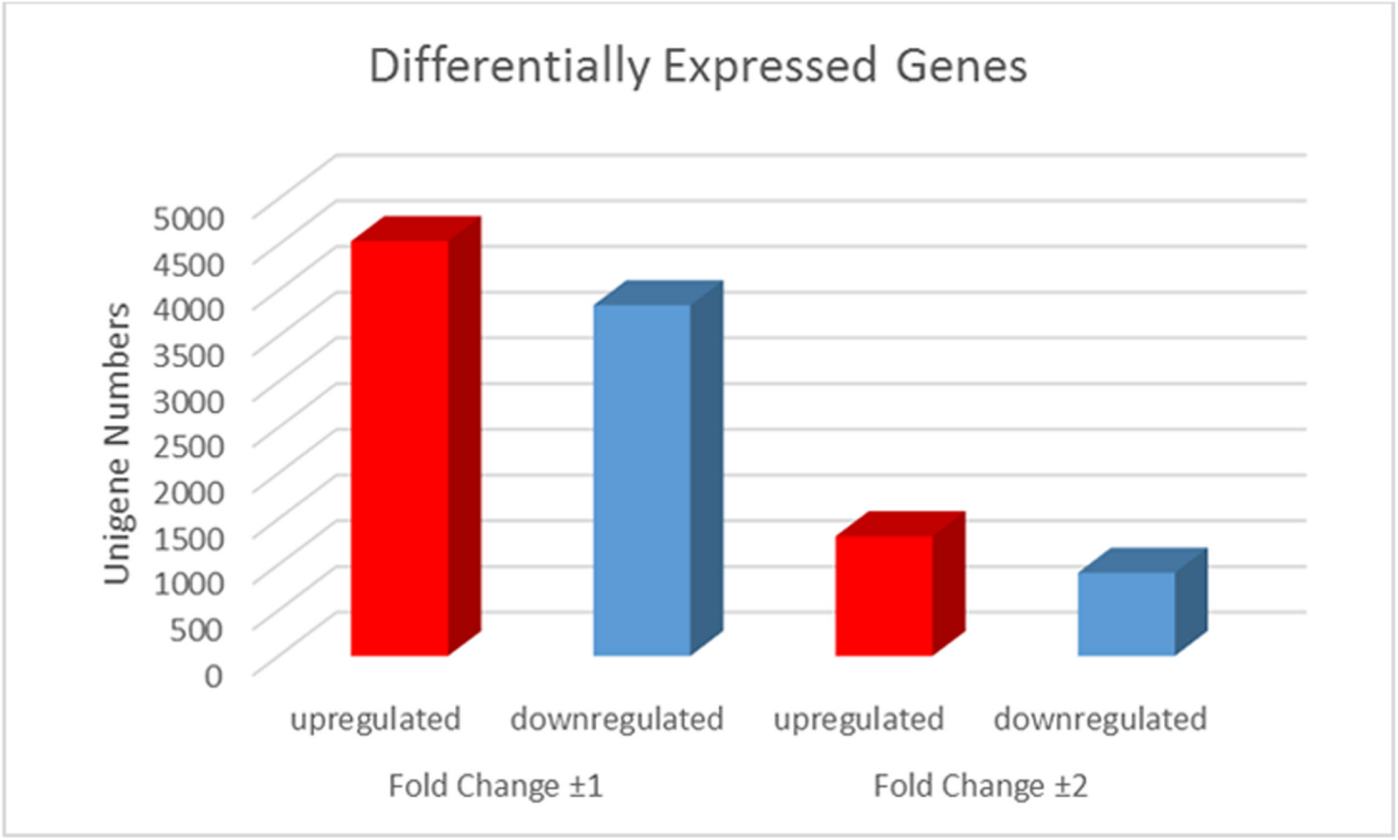
The unigene numbers of Differentially Expressed Genes (DEGs). The red bar is up-regulated genes, and the blue bar is down-regulated genes in Se-treatment vs. control sample respectively.

**Fig 7:**
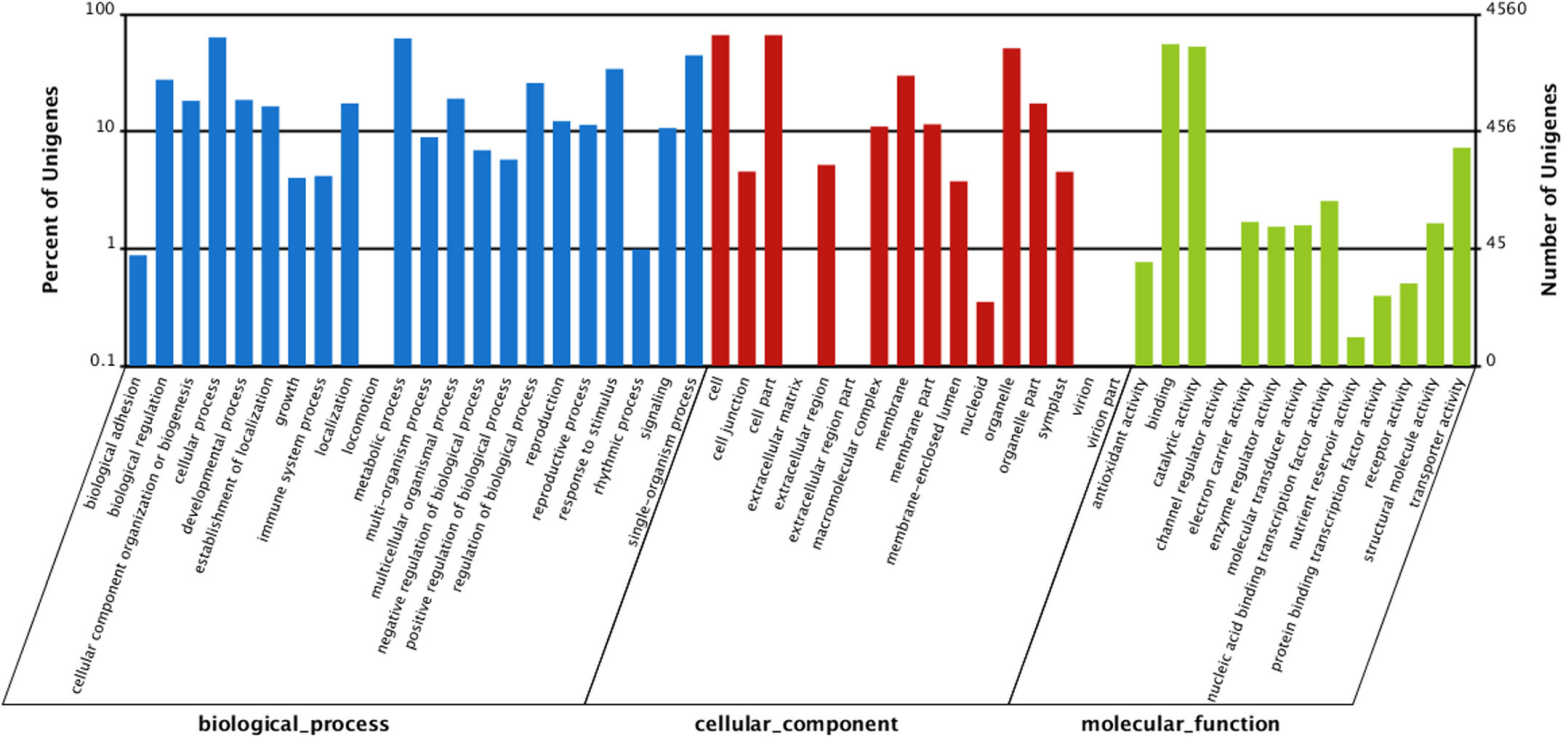
GO enrichment terms as biological process, cellular component and molecular function of differentially expressed genes by Se-treatment.

To understand the effect of Se treatment on gene expression in *A*. *chrysochlorus*, we studied the differentially expressed genes in control and Se-treated callus (**Fig 5 and S3 Table).** Se treatment significantly up-regulated the expression of 4,539 genes (FDR ≤ 0.001, log2 ratio≥ 1). On the other hand, 3,838 genes were downregulated significantly by Se treatment (FDR ≤ 0.001, log2 ratio≥-1) (**Fig 6**). When we adjusted the fold change to ±2, 1311 genes were found to be upregulated and 912 were found to be downregulated. Among these 1311 upregulated and 912 downregulated genes, 663 of upregulated ones and 309 downregulated ones were annotated with nr database (**S3 Table**). We also did gene ontology analysis with the differentially expressed genes to investigate the mechanisms that these genes may belong to (**Fig 7**). These genes were classified into 59 categories (**Fig 7**). In biological process category, cellular process, metabolic process and single cell process ontologies were the most abundant categories with the number of unigenes 2887, 2830 and 2031, respectively. In cellular component terms, cell, cell part and oganelle terms were again the top three categories with 3021, 3021 and 2331 unigenes. Finally, in molecular function category, binding, catalytic activity and transporter activity were the most abundant GO terms with the number of unigenes 2529, 2408 and 331, respectively. According to the KEGG analysis that performed differentially expressed genes, 20 pathways were determined.

## Discussion

Next generation sequencing technologies has been becoming a powerful technology to illuminate the new genes and their involved biochemical pathways in non-model plants. Using RNA-seq, it has become easier to attain the transcriptome data of plant tissues or cells under specific conditions. *Astragalus* plants are valuable and extensively studied because of their properties, however there is little genetic information and transcriptomes. Here we studied the transcriptome of secondary Se accumulator *A*. *chryschlorus* callus tissues under selenium stimuli to bring in new perspectives to selenium accumulation and tolerance. In our study, Illumina Hi-seq sequencing generated totally 9,358,590,780 nucleotides. From these, we obtained 83,273 unigenes with a total of 93,072,751 nt in length with an average length of 1,118 nt for each unigene. Our study identified much more uniqgenes in *A*. *chryschlorus* than that in *A*. *mebranaceus*, a close-related plant species, in which a total of 15,167 contigs, 84,393 singletons, and 9,893 unigenes were assembled [23]. Our results identified many new genes involving in many new biochemical pathways, including Se tolerance and metabolism that have not been illuminated up to now.

### miRNAs may regulate gene expression in response to Se stimuli

**Fig 8:**
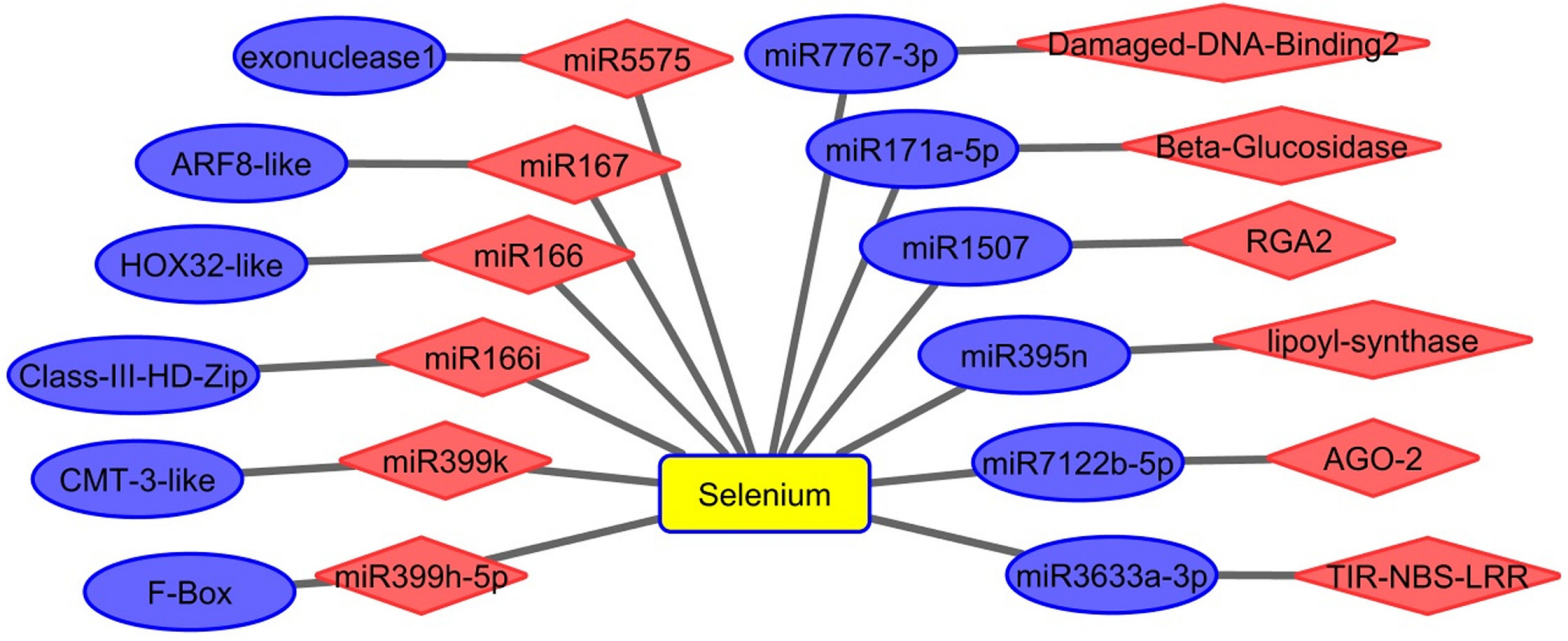
Se affected miRNAs and their targets in *A*. *chrysochlorus* calli based on the degradome data. ARF8 like: auxin response factor 8-like [*Glycine max*], HOX32-like protein: homeobox-leucine zipper protein HOX32-like [*Glycine max*], Class III HD Zip protein: Class III HD-Zip protein [*Medicago truncatula*], CMT-3 like protein: DNA (cytosine-5)-methyltransferase CMT3-like [*Glycine max*], F-Box: F-box protein SKIP2-like [*Glycine max*], Damage DNA Binding 2: protein DAMAGED DNA-BINDING 2 [*Glycine max*], RGA2: Disease resistance protein RGA2 [*Medicago truncatula*], AGO-2: protein argonaute 2-like [*Glycine max*], TIR- NBS-LRR: TIR-NBS-LRR RCT1 resistance protein [*Medicago truncatula*]. Blue color: upregulated, red color: downregulated.

In our previous study, we found that miRNAs, an important gene regulator, play a potential role in *A*. *chrysochlorus* during Se treatment [9]. In this study, we compared the expression of miRNAs, identified in our previous study and miRNA expression, we found the adverse relationship between the expression of miRNAs and their targets (**Fig 8**). During Se treatment, auxin response factor 8-like was upregulated while miR167 was downregulated; The expression of omeobox-leucine zipper protein HOX32-like, Class III HD-Zip protein, DNA (cytosine-5)-methyltransferase CMT3-like, F-box protein SKIP2-like protein was up-regulated by Se exposure but based on the degradome data, their targeting miRNAs, miR166, miR166i, miR399k, miR399h-5p, were down-regulated, respectively. In this study, we found that DAMAGED DNA-BINDING 2, Disease resistance protein RGA2, protein argonaute 2-like, TIR-NBS-LRR RCT1 resistance protein were repressed by Se exposure; in our previous study, we found their targeting miRNAs; miR7767-3p, miR1507, miR7122b-5p and miR3633a-3p were induced by Se treatment, respectively.

**Fig 9:**
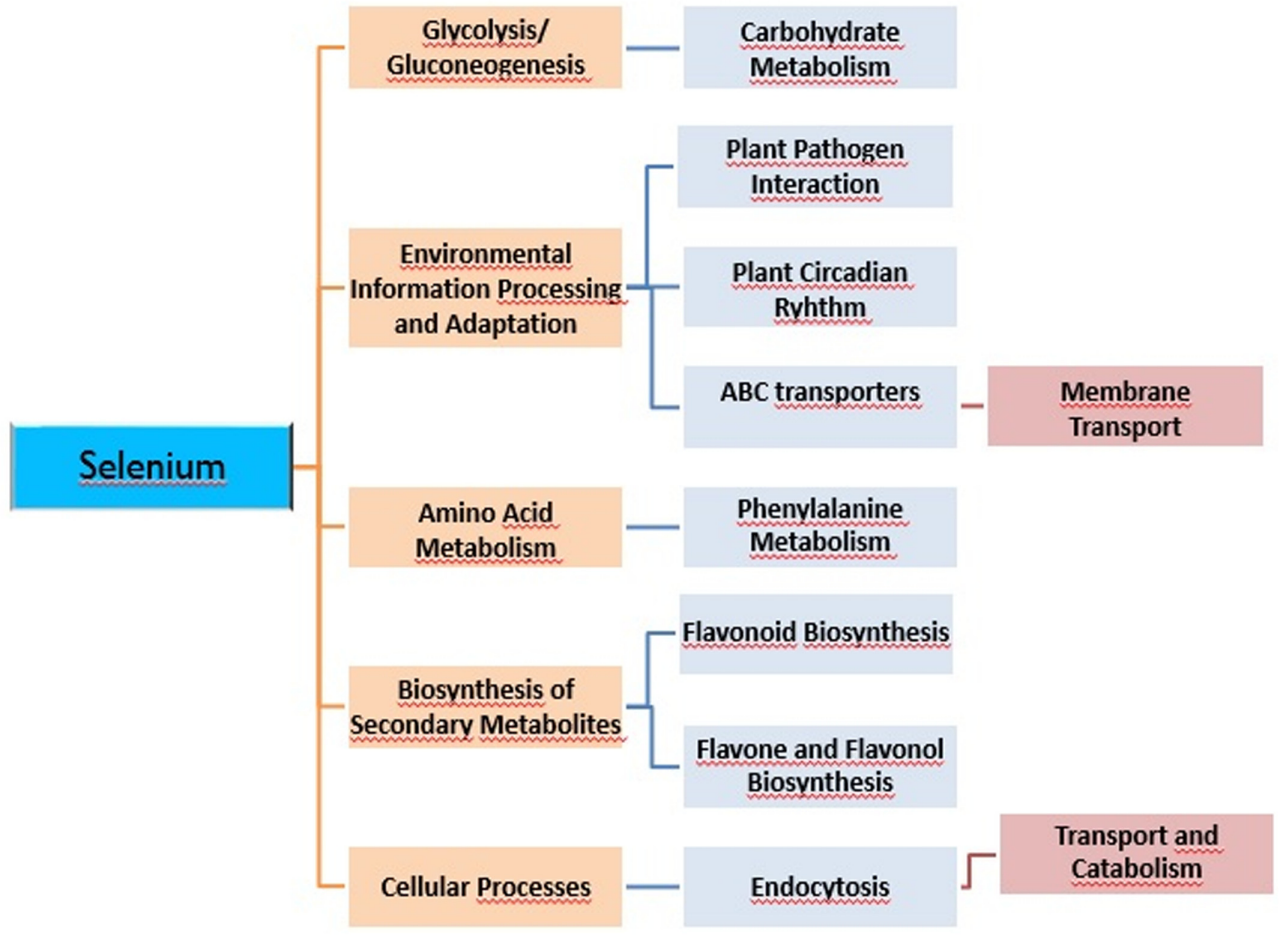
Significantly affected metabolisms of *A*. *chrysochlorus* callus tissues with selenium treatment according to KEGG pathway analysis.

**Fig 10:**
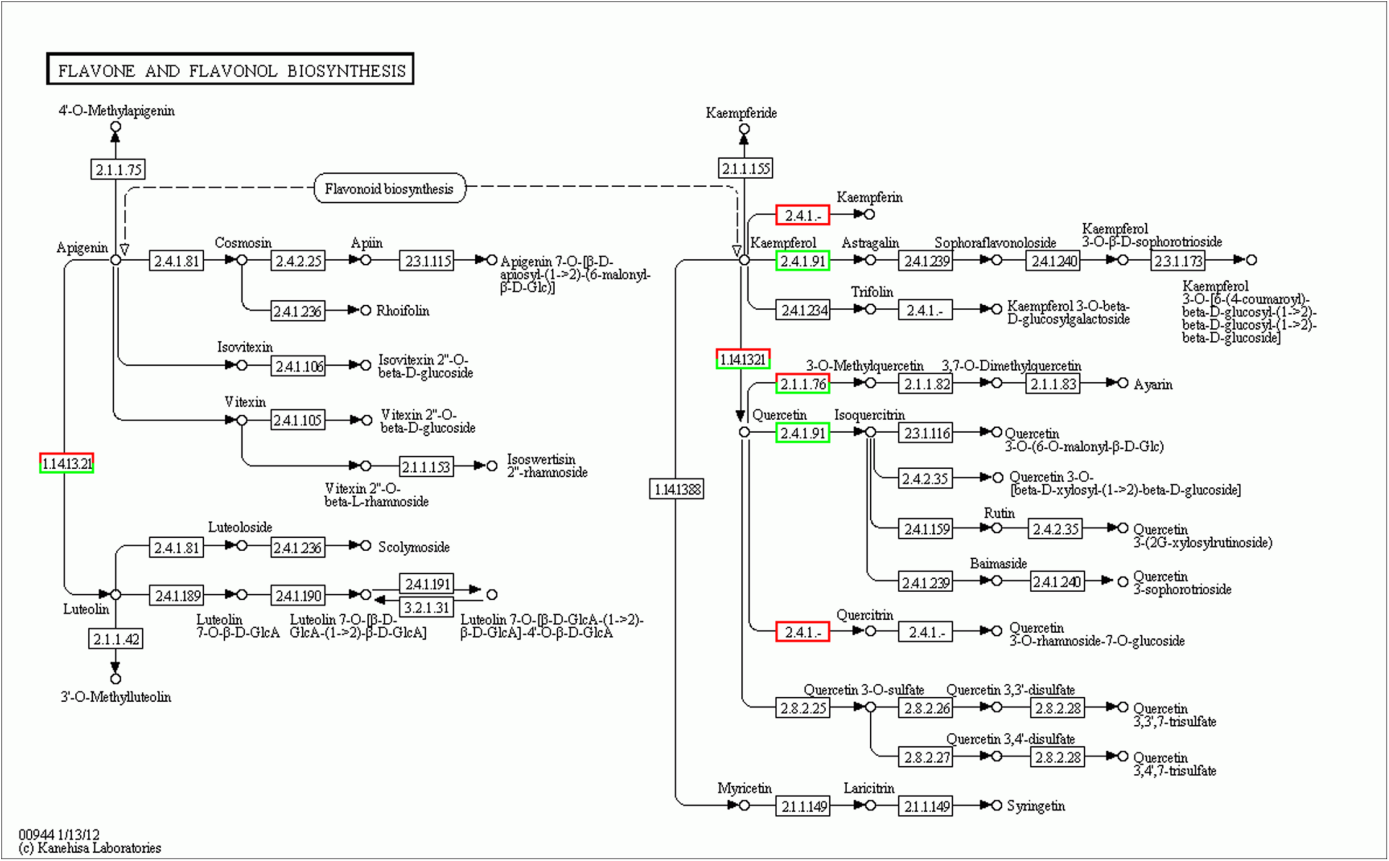
Flavone and Flavonol Biosynthesis pathway involved in Se-treated *A*. *chrysochlorus* calli. Red boxes and green boxes represent up-regulated and down-regulated genes, respectively.

**Fig 11:**
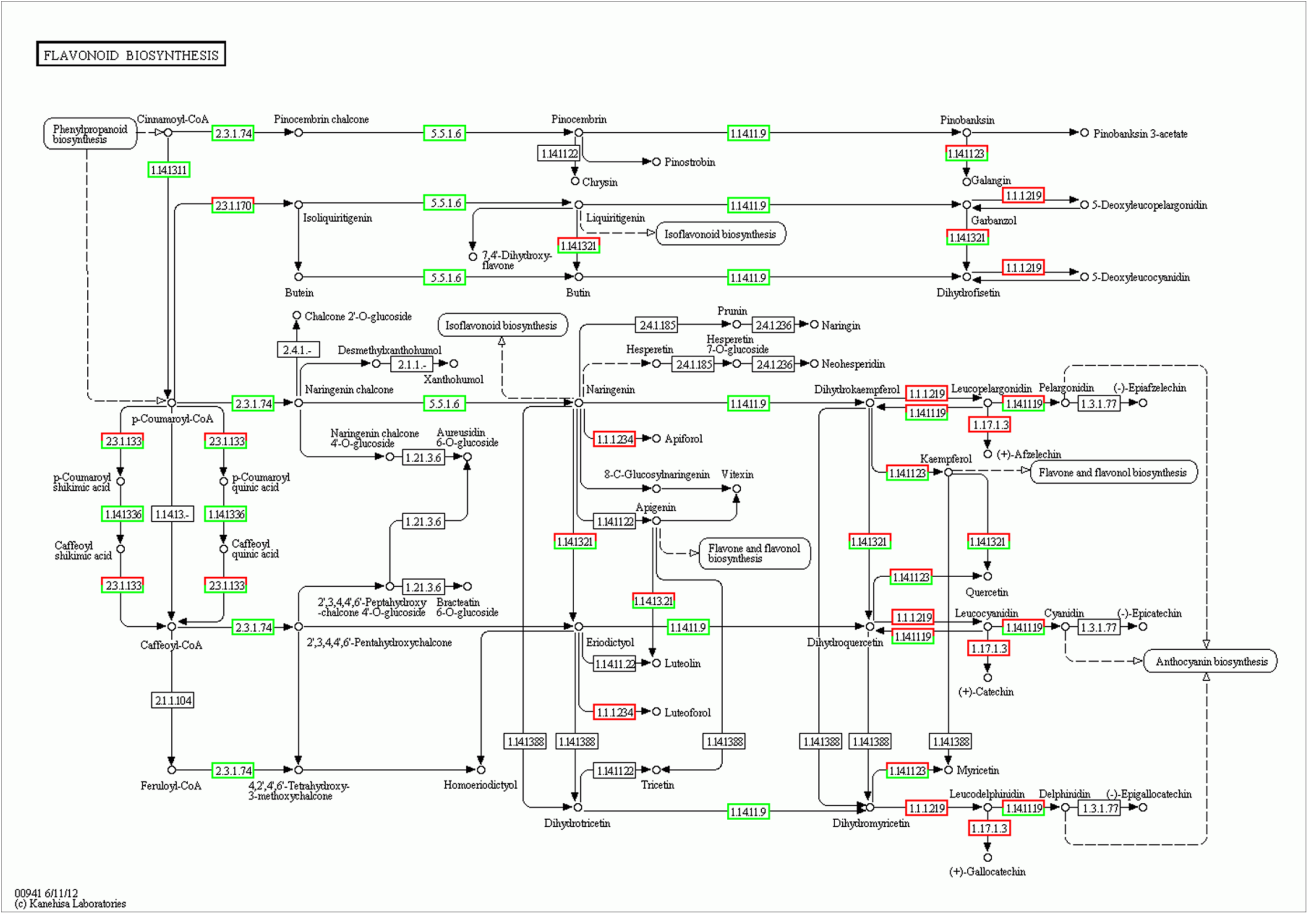
Flavonoid Biosynthesis pathway involved in Se-treated *A*. *chrysochlorus* calli. Red boxes and green boxes represent up-regulated and down-regulated genes, respectively.

Se treatment induced the aberrant expression of many protein-coding genes in *A*. *chrysochlorus* callus tissues. Among the upregulated genes, there are different transcription factors, including TCP13-like (5.64 fold), bZIP transcription factor (2.96 fold), putative transcription factor bHLH041-like (2.87 fold), heat stress transcription factor A-3-like (2.84 fold), trihelix transcription factor GT-3b-like (2.74 fold), B3 domain-containing transcription factor VRN1-like (2.58 fold), probable WRKY transcription factor 32 (2.18 fold), transcription factor bHLH130-like (2.15 fold), transcription factor bZIP (2.06 fold). Another study using *Arabidopsis* found that Se treatment induced the expression of ethylene responsive factors and WRKY family transcription factors, Myb15 and zinc finger proteins [24]. It is known that these kinds of transcription factors are related to plant development as well as plant response to different types of stresses. In our study, we found that selenate treatment resulted in down regulation of ethylene responsive factors. Another upregulated genes were auxin responsive factors such as auxin-responsive protein IAA30-like (3.06 fold), auxin response factor 2-like (2.52 fold), auxin response factor 5-like (2.02 fold). Van Hoewyk et al. [24] found that selenate treatment decreased the level of auxin responsive proteins and this way reduced the plant development. However, in our study, auxin-responsive protein IAA30 was upregulated and it is a transcription factor that represses the early auxin responsive genes at low levels of auxins, even so auxin responsive factor 2-like and auxin responsive factor 5-like proteins seem to be upregulated. Auxin responsive factor 2 is known to be transcription activator/repressor that activates/represses early auxin responsive genes. These early auxin responsive genes are related to plant growth and development [25]. However auxin responsive factor 5 is known to be a transcription activator. ABC transporter family proteins are known to be involved in growth, nutrition, development, abiotic stress response, and environment-plant interaction [26]. In our study ABC transporter family B member, B member 1-like, B member 1-like isoform 2, B member 11-like, B member 28-like proteins, ABC transporter family C member, C member 5-like, ABC transporter family G member, G member like-28 were all upregulated between 2 to 11 folds. On the other hand, ABC transporter family A member 2 and member 7, family G member 5 were downregulated between 2,8 and 2.03 folds. ABC transporter family proteins are involved in detoxification and transport processes in plants [26] and when considered from this aspect, it is not surprising to detect these genes in cells after selenium treatment. Se and selenocompounds could be toxic for most the plants and *Astragalus* plants are known to tolerate high levels of Se, also in our previous study, *A*. *chrysochlorus* was found to be secondary accumulator. Another interesting finding is the alteration in expression of disease resistance genes, cc-nbs-lrr (coiled-coil nucleotide-binding site leucine-rich repeat) resistance genes and tir-nbs-lrr (Toll/interleukin-1 receptor nucleotide-binding site leucine-rich repeat) resistance genes. These resistance proteins are involved in pathogen recognition [27]. It was found that while some of these resistance genes were upregulated, some of them were downregulated. The majority of the downregulated resistance genes were tir-nbs-lrr and cc-nbs-lrr genes. However among the upregulated ones, there were multidrug resistance ABC transporter gene, TMV resistance gene besides tir-nbs-lrr and cc-nbs-lrr genes. A study showed that several disease and stress induced proteins were upregulated in *Arabidopsis* [24]. It seems that calcium related genes were upregulated in Se treated callus tissues in *A*. *chrysochlorus*. The upregulated genes were annotated with calcium-dependent protein kinases, calcium-dependent protein kinase 20-like. These types of plant kinases bind calcium ions and phosphorylate metabolites which are related to many cellular mechanisms such as hormone response and stress signaling pathways [28]. Other genes related to calcium were type IIB calcium ATPase, calcium-transporting ATPase 4 plasma membrane-type-like, calcium-transporting ATPase 9 plasma membrane-type-like proteins. These proteins also are involved in adaptation to stressors by altering the calcium concentrations [29]. According to the Gene Ontology and Kyoto Encyclopedia of Genes and Genomes database pathway analysis, Se affected metabolic pathways and genes such as ABC transporters, plant pathogen interaction, phenylalanine metabolism, biosynthesis of secondary metabolites, flavone and flavonol biosynthesis, flavonoid biosynthesis, plant circadian rhythm, glycolysis/gluconeogenesis and endocytosis genes (**Fig 9**). The metabolites that may help to adapt the environment were thought to be produced by flavone and flavonol biosynthesis, flavonoid biosynthesis pathways (**Figs 10** and **11**). It seems that in flavon and flavonol biosynthesis pathway, flavonol-3-O-rhamnosyltransferase was upregulated and flavonol 3-O-glucosyltransferase was downregulated by Se treatment (**Fig 10**). In flavonoid biosynthesis pathway, chalcone synthase, chalcone isomerase, trans-cinnamate 4-monooxygenase, naringenin 3-dioxygenase and coumaroylquinate (coumaroylshikimate) 3'-monooxygenase were determined as downregulated while bifunctional dihydroflavonol 4-reductase/flavanone 4-reductase and leucoanthocyanidin reductase were found to be upregulated (**Fig 11**). These metabolic pathways are all somewhat related to environmental adaptation and stress response [30]. Basically, in our previous study, it was determined that plant pathogen interaction pathway is affected by miRNAs under Se stress [9]. Here in this study, we confirm that the expression of the genes in this pathway were affected by Se. Besides this fact, it was an intriguing finding to detect other pathways and genes associated with responses to environmental stimuli.

In conclusion, we assembled for the first time the transciptome of *A*. *chrysochlorus de novo*. We also studied the expression difference of genes responsive to Se exposure. Our results showed that *A*. *chrysochlorus* sequences are mostly similar to *G*. *max* (43.1%) and *M*. *truncatula* (39.7%). We detected total of 1,311 genes were upregulated by Se treatment awhile 912 of genes were down-regulated genes. ABC transporters, plant pathogen interaction, biosynthesis of secondary metabolites genes are affected by Se stimuli in *A*. *chrysochlorus*. We believe that our results enable a new intellection about elucidating the cellular processes about Se accumulation and tolerance in plants.

## Supporting Information

S1 Fig Sequence size of unigenes. (XLSX)

S1 Table Coverage of control library sequencing. (XLSX)

S2 Table Coverage of selenium library sequencing. (XLSX)

S3 Table Differentially expressed genes of Se-treated and untreated *Astragalus chrysochlorus calli*. (XLSX)
